# Effect of Malaria and Geohelminth Infection on Birth Outcomes in Kumasi, Ghana

**DOI:** 10.9734/IJTDH/2014/7573

**Published:** 2014

**Authors:** Ntui N. Asundep, Pauline E. Jolly, April P. Carson, Cornelius A. Turpin, Kui Zhang, Nana O. Wilson, Jonathan K. Stiles, Berhanu Tameru

**Affiliations:** 1 Department of Epidemiology, School of Public Health, University of Alabama at Birmingham, Birmingham, AL 35294, USA; 2 Komfo Anokye Teaching Hospital, Kumasi, Ghana; 3 Department of Biostatistics, School of Public Health, University of Alabama at Birmingham, Birmingham, AL 35294, USA; 4 Department of Microbiology, Biochemistry and Immunology, Morehouse School of Medicine, Atlanta, GA 30310, USA; 5 Center for Computational Epidemiology, Bioinformatics and Risk Analysis (CCEBRA), Tuskegee University, Tuskegee AL 36088, USA

**Keywords:** Geohelminths, malaria, pregnancy outcomes, Kumasi

## Abstract

**Aim:**

In 2005, the Ghana Health Service mandated malaria and helminths chemoprophylaxis during antenatal care visits. The aim of this study was to investigate the prevalence of malaria and helminth infections and their relationship with adverse birth outcomes (low birth weight, stillbirth, and preterm) following the implementation of these treatments.

**Study Design:**

A quantitative cross-sectional study.

**Method:**

The study was conducted on 630 women presenting for delivery in the Komfo Anokye Teaching Hospital and the Manhyia District Hospital from July to November 2011. Socio-demographic information and medical and obstetric history were collected. Laboratory analyses for the presence of malaria and helminths were performed. Association of malaria and helminths with birth outcomes was assessed using logistic regression to obtain odds ratios (ORs) and 95% confidence intervals.

**Results:**

The prevalence of malaria, helminths and adverse birth outcomes was 9.0%, 5.0% and 22.2%, respectively. Compared with women who received malaria prophylaxis, women without malaria prophylaxis were two times more likely to have malaria infection (aOR = 2.1; 95% CI = 1.06-4.17). Women who were not screened for helminths were twice as likely to be infected with helminths (aOR = 2.4; 95% CI = 1.15-5.12) than women who were screened for helminths. For women infected with hookworm or *Schistosoma mansoni*, the odds of having an adverse birth outcome (aOR = 3.9; 95% CI = 1.09-14.20) and stillbirth (aOR = 7.7; 95% CI = 1.21-36.38) were greater than for women who were not infected.

**Conclusion:**

The prevalence of malaria, helminths and adverse birth outcomes was lower than previously reported 9.0% vs. 36.3, 5.0% vs. 25.7 and 22.2% vs. 44.6, respectively. Helminth but not malaria infection was found to be significantly associated with adverse birth outcomes.

## 1. Introduction

Malaria and helminths are endemic in most sub-Saharan African countries. A study in Nigeria demonstrated that over 45% of Plasmodium infected near-term mothers also had intestinal helminths [1]. These infections have been associated with low haemoglobin (Hb) levels and iron/folate deficiency anemia especially in primigravid women [1]. Fifty percent of pregnant women in developing countries are often at a high risk of experiencing adverse birth outcomes due to iron/folate deficiency [1,2]. Iron deficiency anemia during pregnancy is associated with lower iron stores in the fetus, thus there is need for iron supplementation during pregnancy [3,4]. Moderate to severe anemia is associated with an increased risk of low birth weight and preterm delivery [4].

Pregnant women living in malaria endemic areas of Africa are at an increased risk of *Plasmodium falciparum* infection [3,5]. Malaria in pregnancy is a major cause of maternal and fetal morbidity and mortality [2,6]. The prevalence of malaria is influenced by a number of factors such as maternal age, parity, use of prophylaxis, nutritional status, host genetics, parasite genetics and transmission rate [7]. Compared to other malaria parasites, *P. falciparum* has been found to be a stronger contributor to pregnancy anemia especially in nulliparous women [4,6]. Malaria is endemic in Kumasi, Ghana and the predominant malaria parasite is *P. falciparum*. The area is also endemic for both hookworm and schistosomiasis [5]. The combined effects of poor nutrition, inadequate potable water supply, poor sanitation and perennial malaria transmission contribute significantly to pregnancy anemia and consequently adverse birth outcomes [3,8]. Prevalence of hookworm infection among pregnant women in sub-Saharan Africa is estimated at 32% [9]. Studies in Nepal suggested that 54-58% of moderate to severe anemia in pregnancy was due to hookworm [4]. The role of malaria, hookworm and trichuris infections in pregnancy anemia has been established [8]. The helminth parasites cause blood loss that sometimes induce pregnancy anemia [10]. Both synergistic and antagonistic effects due to malaria and helminths co-infections have been suggested. While one study reported a positive association between *Ascaris lumbricoides* and *P. falciparum* [11], another study reported suppression of malaria infection in patients with *A. lumbricoides* [12]. The synergistic action from the co-infection was linked to the fact that helminth parasites induce elevated T-helper-2 (Th2) cytokines and down regulate Th1 type immune response increasing susceptibility to other infections including malaria [13].

A 44.6% prevalence of adverse birth outcomes, 36.3% prevalence of malaria and 25.7% helminth prevalence has been previously reported among women with uncomplicated pregnancy in Kumasi [6]. Acknowledging the impact of malaria and helminths in pregnancy, Ghana Health Service in 2005 modified its antenatal care (ANC) protocol to include mandatory chemoprophylaxis for malaria and helminths during ANC visits. During ANC visits women are expected to receive at least three doses of Sulfadoxin-pyrimethamine (SP) [14]. The first dose is given any time after the first trimester and subsequent doses are given at least one month apart as directly observed therapy (DOT) [15,16]. For women deficient in glucose-6-phosphate dehydrogenase (G6PD), a single dose of an artemisinin combination therapy (ACT) or its derivative is recommended. A single dose of anthelmintic (mebendazole 500mg) is also recommended during the 2nd and 3rd trimesters [17].

The study that reported a 44.6% prevalence of adverse birth outcomes was done in 2006 and published in 2010 [18]. In 2006 the changes recommended by the Ghana Health Service in 2005 had not taken full effect. Therefore, this is the first study conducted to investigate the prevalence of malaria and helminth infections among pregnant women and association of these parasitic infections with adverse birth outcomes since the full implementation of the Ghana Health Service protocol.

## 2. Materials and Methods

### 2.1 Study Setting

A quantitative cross-sectional study was conducted in Kumasi, the capital of the Ashanti Region of Ghana. Kumasi has an estimated daytime population of 1.7 million people (Kumasi Metro Profile, 2011 estimate; Unpublished; Joana Tawia Burgesson). Kumasi has 5 health sub-metros (Asokwa, Bantama, Manhyia North, Manhyia South and Subin). This study was conducted in two health facilities in Kumasi, namely, the Komfo Anokye Teaching Hospital (KATH) and the Manhyia District Hospital (a tertiary and secondary hospital, respectively) from July to November 2011. KATH is a referral hospital that provides most ANC, labor and delivery services and serves the entire Ashanti Region and the bordering Brong-Ahafo, Central, Eastern and Western Regions. Manhyia District hospital covers Manhyia North and South and serves 34.6% of the Kumasi population. Kumasi has perennial malaria transmission (mainly *P. falciparum*) with two rainy seasons lasting from April to June and September to October [19]. Kumasi is also endemic for helminth infections [5].

### 2.2 Participant Recruitment

Eligible participants were pregnant women, 19 years and older, who resided or moved to Kumasi within 1-2 months following conception. Women who presented for delivery at the two study sites within the study period, met the eligibility criteria and consented to the study, were recruited. Women with spontaneous, vaginal, singleton deliveries, without complications were eligible for enrollment in this study. Informed consent was obtained from all study participants. Data for 630 participants were used for these analyses. Trained study personnel administered the questionnaire to the participants 1-2 hours after delivery in a private area. The questionnaires were reviewed for completeness.

### 2.3 Study Instrument

The details of the study instrument and data collected have been described elsewhere [20]. Information obtained included: 1) socio-demographics; 2) malaria prophylaxis; 3) use of insecticide treated bed nets; 4) duration of pregnancy; and 5) helminth screening and prophylaxis. Data on intermittent preventive therapy (IPT) with sulfdoxine-pyrimethamine (IPT-SP) and haemoglobin concentration were abstracted from the women's antenatal booklets while data on pregnancy outcomes were ascertained from maternity records.

### 2.4 Sample Collection and Analyses

Five to ten grams of stool were collected from the women and preserved in a 30 ml tube containing 15 ml of 10% formalin. The samples were agitated several times, assigned participant's unique identification numbers (PID) and stored at room temperature. Samples were analyzed at the end of the study in the parasitological laboratory of the outpatient unit at KATH. Determination of helminths was done using the Formalin-Ether concentration method. Samples were processed in conformity to the standard protocol [21,22]. The preparations were examined microscopically and helminth types were documented.

Peripheral blood (5 ml) was drawn by venipuncture soon after delivery into a vacutainer tube without anti-coagulant to facilitate clotting and labeled with the participant's ID number. The clotted blood samples were spun at 4000 rpm at 4ºC and the extracted serum frozen at -20ºC until analyzed for malaria antigen using the Malaria Antigen Celisa kit (Cellabs, Brookvale, Australia). The Celisa kit is a monoclonal antibody assay based on the detection of malaria antigen (HRP II) a highly sensitive marker for *P. falciparum* malaria in blood and serum. The assay detects the merozoite antigen that circulates in the blood and a positive result is suggestive of current or very recent infection. The ELISA test results were considered positive when the optical density was >0.2 over the negative control. The assay has been shown to detect *P. falciparum* parasitaemia as low as 0.001% with a sensitivity of approximately 98.8% and specificity of 100% [23].

### 2.5 Definition of Variables

Adverse birth outcome in this study includes any of the following outcomes; i) low birth weight defined as birth weight < 2,500 g; ii) preterm delivery (<37 weeks of gestation); iii) definition of stillbirth has been adapted for this study to mean the death of a fetus prior to, during or soon after delivery, but before discharge from the hospital (since we could not have access to the new mothers 12h after delivery). At the study site women are monitored for about 1-2h in the maternity ward after delivery. They are then taken to the “Lying Inn Ward” for further observation for 2–4h before being discharged from the hospital. *Malaria infection:* Presence of malaria antigen in the peripheral blood at the time of delivery. *Helminth infection*: Presence of helminth eggs or larvae in stool at the time of delivery. *Co-infection:* Presence of both malaria antigen and any helminth infection at delivery [18]. *Severe/moderate anemia:* haemoglobin level <8 g/dL of blood, *mild anemia:* haemoglobin levels ≥ 8 and < 11 g/dL of blood [24].

### 2.6 Data Analysis

The data were single-entered in a Microsoft^®^ Access 2010 database and imported into SAS for coding and analysis. Cochran-Mantel Haenszel Chi-square or Fisher's exact tests were used to assess the associations between participants' characteristics by infection status, and association of ANC services received and adverse birth outcomes by infection status. Stratum specific estimates to assess effect modification by study sites were obtained. To access the validity of the self-reported data, Kappa coefficients were calculated to test the agreements between self-reported malaria prophylaxis and data abstracted from maternal antenatal record. Values of 0.4-0.6 indicate moderate, 0.6-0.8 substantial and 0.8 or higher as excellent agreement [25]. Logistic regression models were used to examine the association between malaria and helminth infection with adverse birth outcomes: a) a crude model examined the association of malaria and helminth infection with any adverse birth outcome and type of adverse birth outcomes (low birth weight, preterm and stillbirth); b) a final multivariable logistic model examined the association of malaria and helminths with adverse birth outcomes while adjusting for age, parity, human immunodeficiency virus (HIV) status and haemoglobin level. Crude and adjusted odds ratios (ORs and aORs) with 95% confidence intervals (CI) and p-values were calculated. The change-in-estimate criteria were used to select potential confounders. A variable was considered a confounder if the change in estimate from the crude and adjusted model was at least 10 % [26]. All tests were two sided and p-values ≤ 0.05 were considered statistically significant. All analyses were done using SAS^®^ 9.3 (SAS Institute, Cary, NC, USA).

## 3. Results

### 3.1 Participant's Characteristics and Infection Status

Participation rate in the study was 99.7%. Mean age ± standard deviation (SD) was 27.9±5.7 years and ranged from 19-48 years. Approximately 38% (239/630) of participants were less than 25 years. Sixty-eight percent (427/630) of the women had a monthly income of less than GH¢500.00 (US$ 333.3). Twenty-one percent (130/629) were unemployed while 69.0% (435/629) were self-employed. Approximately 58% (252/435) of these self-employed were traders and 30.6% (133/435) were hairdressers or seamstresses. Women who were infected with malaria were more likely to be single and unemployed. Forty-three percent of the participants attended ANC more than the required minimum of 8 visits (Ghana's standard). Single women (15.4%; 16/104; P = .04) and unemployed women (13.9%; 18/130; P = .04) were disproportionately infected with malaria than women who were married and employed. The prevalence of helminths infection was also higher for women who earned less than GH¢500.00 (6.3%; 27/427) compared to those who earned more than GH¢ 500.00 (2.8%; 5/176) (Table 1).

### 3.2 Infection Status and Predictors of Malaria and Helminth Infections

Participants' malaria and helminth infection status and prevention services received during ANC visits are presented in Table 2. The prevalence of helminths and malaria in the study population was 5.0% and 9.0% respectively. Helminths that were identified were *Dicrocoelium dendriticum* (liver fluke)*, Strongyloides stercoralis, Schistosoma mansoni* (blood fluke) and *Ancylostoma sp*. (hookworm). The prevalence of malaria was higher (15.3%) in women who did not receive malaria prophylaxis than those who did (8.4%). Women who did not receive malaria prophylaxis were two times more likely to be infected with malaria (aOR=2.1; 95% CI=1.06-4.17; *P=.03*) compared with women who received a complete regimen of malaria prophylaxis. The prevalence of helminth infection was also higher for women who were not screened for helminths (8.6%) than women who were screened and received chemoprophylaxis (4.0%). For unscreened women, the risk of helminths infection was two times higher compared with women who were screened (aOR=2.4; 95% CI=1.15-5.12; *P=.02*) after controlling for the study site (Table 2).

A higher proportion of women with low haemoglobin (severe/moderate anemia) tended to be infected with malaria and helminths than women with normal haemoglobin concentration, but this trend was not statistically significant (aOR = 1.7, 95% CI = .68-4.00, *P = .27*; aOR = 1.7, 95% CI = .53-5.17, *P = .38*), respectively (Table 3).

### 3.3 Infection Status and Adverse Birth Outcomes

Twenty-two percent of the women experienced an adverse birth outcome. The prevalence of adverse birth outcomes was higher in women infected with malaria (11.4%) or helminths (7.1%) compared with uninfected women (8.4% and 4.5%, respectively). Though not statistically significant, women with malaria or helminths were 40% and 60%, respectively, more likely to experience an adverse birth outcome compared with women without these infections (Table 3). However, women infected with helminths associated with blood loss (hookworm or blood fluke) were three and a half times more likely to experience an adverse birth outcome (OR = 3.6; 95% CI = 1.03-12.63), four times more likely to have a low birth weight baby (OR = 4.1; 95% CI = 1.02-16.17), and six times more likely to have a stillbirth (OR = 6.0; 95% CI = 1.5-24.00). After adjusting for confounders (age, parity, HIV, and haemoglobin level), women infected with hookworm or blood fluke were four times more likely to experience an adverse birth outcome (aOR = 3.9; 95% CI = 1.09-14.20) and eight times more likely to have a stillbirth (aOR = 7.7; 95% CI = 1.2-36.38) compared with women who were not infected with these helminths. In both the crude and adjusted models, malaria and malaria-helminth co-infections were not statistically significantly associated with adverse birth outcomes (Table 4).

## 4. Discussion

Malaria and helminth infections are of serious public health concerns in developing countries especially in countries of sub-Saharan Africa. This study found a lower prevalence of malaria (9.0%), helminths (5.0%) and adverse birth outcomes (22.2%) among women in Kumasi in 2011 than previously studied in 2006 (reported 2010) [6]. This reduction could be due to the fact that the changes to the ANC protocol initiated by the Ghana Health Service in 2005 were not fully implemented in 2006 as they were by 2011 when this study was conducted. The finding from this study partly corroborates that of another study conducted in Uganda that reported an association between malaria and helminths with adverse birth outcomes [8]. In this study, only helminths that cause blood loss were associated with adverse birth outcomes. The data did not support the association of adverse birth outcomes with malaria. This could be due to the small case numbers or the high uptake of IPT (SP or ACT). The high rate of IPT uptake could be attributed to the fact that the medication is free, and is administered as DOT, and that when a woman initiates ANC, the date of the next visit is indicated in the maternal antenatal booklet. As previously reported, the ANC services received in Kumasi are dependent on the time of ANC initiation and the frequency of ANC attendance [20]. It is intriguing and worth pointing out that malaria infection was lowest in women with one dose of SP compared with women with three doses of SP. The reasons are not obvious and warrant further investigation. It is common practice that pregnant women receive folic acid as nutritional supplement. Concomitant administration of SP and folic acid could be contributing to development of resistance to SP.

Though malaria infection was not directly associated with adverse birth outcomes in this study, a high proportion of women with malaria had severe anemia. Malaria especially *P. falciparum* has often been associated with anemia in developing countries [4] and severe anemia is a predictor for adverse birth outcomes [20]. A review of the impact of malaria related anemia among pregnant women in sub-Saharan Africa suggested that over a quarter of cases of severe anemia were attributable to malaria infection [27]. The malaria pathogen acts by directly destroying erythrocytes while malaria pigments and pro-inflammatory mediators may also inhibit erythropoiesis [28]. Hypersplenism, sequestration of red blood cells and phagocytosis of parasitized and un-parasitized red blood cells have also been suggested as possible mechanisms by which malaria causes anemia [29]. Though malaria has often been associated with adverse birth outcomes, our data did not support this association. More than 84% of the women received at least one dose of SP (mean dose = 2.1) given as DOT to ensure compliance [20]. The timing of the IPT regimen could have played a role in controlling adverse birth outcomes especially during growth and development (2nd trimester of pregnancy). Other factors such as blood aflatoxins B1 levels could predispose pregnant women to pregnancy anemia thereby contributing to adverse birth outcomes. An association between aflatoxins B1 blood levels and anemia and adverse birth outcomes in the same study area has been previously reported [30].

The results from this study suggest that infection with any helminth was marginally associated with low birth weight. However, infection with any of the helminths that cause blood loss (hookworm or blood fluke) was significantly associated with stillbirth and low birth weight. A prior study in the same geographic area did not find any association between helminths infection and stillbirth [6]. The fact that the study did not categorize these helminths according to their association with blood loss as was done in this study could explain the absence of such an association. This study corroborates the result of a study in the Amazon region of Peru that found an association between helminths and stillbirth [31]. Schistosomiasis and hookworm cause blood loss [3] and it is recommended that pregnant women receive antihelmintics after the first trimester especially in areas with >20–30% prevalence of hookworm and prevalent anemia [32]. Hookworm releases anti-coagulants and cause mechanical lacerations to the walls of the small intestines leading to blood loss in the stool and depletion of iron store. Intestinal blood loss, mal-absorption and reduced appetite further exacerbate iron deficiency anemia in pregnant women [3,29]. While the drugs of choice (albendazole and mebendazole) are effective against hookworm they may not be as effective for schistosomes. A clinical trial on the use of mefloquine with activity against *S. haematobium* as IPT in Gabon may provide a solution and reduce the reliance on praziquantel. In rodent models, mefloquine has been shown to have considerable activity against *S. mansoni* and *S. japonicum* the causative agents of intestinal schistosomiasis [33]. Seventy-six percent of the participants were screened for intestinal helminths but only 23.6% reported receiving antihelmintics. This low reportrate could be due to the fact that these women were treated but were not informed of the purpose of the treatment. There is also the possibility of self-medication; albendazole the drug of choice is readily available without prescription. The low sensitivity of the Direct Wet Mount method that is routinely used in Ghana for parasitological screening could produce false negative results with the consequence that no treatment is administered. According to the revised ANC protocol, these women should have received 2 doses of antihelmintics one each during the 2^nd^ and 3^rd^ trimesters [17] if they attended ANC regularly. The inclusion of antihelmintics as DOT as is the case with SP in the revised Ghana protocol could save time for the women and also the resources that go into stool examination. If the use of IPT as DOT led to a 26% reduction in the prevalence of malaria then administration of antihelmintics as DOT could positively impact the fight against helminths in pregnancy.

Brooker et al. in their review summarized that deworming and iron supplementation would have a greater impact on haemoglobin level than deworming alone especially in populations with diets low in bioavailable iron [4,27]. In a study in the South West Province of Cameroon, 69% of women who were given pyrimethamine, iron and folate but not mebendazole had maternal anemia at mean gestational age of 24 weeks [2]. Despite the low prevalence of helminths in this study (5.0%), their association with adverse birth outcomes underscores the serious adverse health impact of these parasites. The high prevalence of adverse birth outcomes observed despite the low prevalence of malaria and helminth infections in the study population suggest that there could be other factors such as haemoglobinopathies (sickle cell, thalassemia, G6PD deficiency) genetics, and environmental factors that are yet to be investigated. Nutritional status during pregnancy, metabolic diseases and HIV status are among many other factors that could adversely impact pregnancy outcomes. HIV was not an important factor in this study as the prevalence in the study population was low (0.01%; 8/630). However, we adjusted for HIV in the multivariable analyses.

This is the first study to investigate the prevalence of both malaria and helminths in this region of Ghana since the full implementation of the changes made in the ANC protocol. This study demonstrates a lower prevalence of both malaria and helminth infections and a reduced prevalence of adverse birth outcomes among pregnant women in Kumasi. It is tempting to speculate that the changes made to the Ghana ANC delivery system are responsible for this reduced prevalence. Since we did not quantify the level of *P. falciparum* infection, it is possible that the lack of association of malaria and adverse birth outcomes could be due to mild infections. Also, it is possible that an episode of malaria early in the pregnancy could impact the outcome that could not be detected at the time of the study. However, we recommend that more studies be conducted in both urban and rural areas to determine the true prevalence of these infections in pregnant women and their association with adverse birth outcomes. The relatively low number of cases should be taken into consideration in interpreting the results. However, the small cell counts was not a problem since the adequacy of the sample size is determined by the across strata sample size [23] and the study had sufficient power. Since our cross-sectional study design limits our ability to draw causal or temporal associations a longitudinal study to establish causality and temporality need to be conducted. There is always the problem of recall bias in collecting data with the use of questionnaire. However, the substantial agreement (kappa = 0.65) between self-reported use of IPT and the data abstracted from maternal antenatal record indicates that the effect of recall bias could be minimal. This study could be used as a proxy for the evaluation of the effectiveness of the changes made to the ANC protocol because the indicators (malaria and helminth infection status) were objectively assessed. Also, the number of doses of IPT received was obtained from maternal antenatal records and not from participants. Thus, we consider these findings to be representative of women with uncomplicated pregnancies in Kumasi.

## 5. Conclusion

The prevalence of malaria, helminths and adverse birth outcomes was lower in 2011 than previously studied in 2006 (9.0% vs. 36.3%, 5.0% vs. 25.7% and 22.2% vs. 44.6%, respectively). Helminth infection, but not malaria infection, was statistically significantly associated with adverse birth outcomes.

## Figures and Tables

**Table 1:** Infection status by participant's characteristics, Kumasi 2011 (Row %)

Characteristics	N = 630		Malaria N = 57		Helminths N = 32	

	N	%	N	%	N	%
Age group						
≤ 25	239	37.9	25	10.5	12	5.0
26 - 35	328	52.1	25	7.6	17	5.2
> 35	63	10.0	7	11.1	3	4.8
Level of education						
Primary/None	196	31.1	21	10.7	14	7.1
Junior secondary	271	43.0	27	10.0	14	5.2
Senior secondary	92	14.6	6	6.5	3	3.3
Vocational/University	71	11.3	3	4.2	1	1.4
Marital status*						
Single	104	16.5	16	15.4	3	2.9
Married	450	71.4	34	7.6	26	5.8
Living as married	76	12.1	7	9.2	3	4.0
Employment a*						
Unemployed	130	20.7	18	13.9	10	7.7
Employed	64	10.2	2	3.1	2	3.1
Self-employed	435	69.2	37	8.5	20	4.6
Religion						
Christianity	460	73.0	43	9.4	22	4.8
Islam	162	25.7	14	8.6	10	6.2
None	8	1.3	0	0.0	0	0.0
Income (Cedis)						
< 500	427	67.8	36	8.4	27	6.3
500 - 2000	176	27.9	17	9.7	5	2.8
> 2000	27	4.3	4	14.8	0	0.0
Parity						
1	224	35.6	22	9.8	14	6.3
2 - 5	385	61.1	32	8.3	16	4.2
> 5	21	3.3	3	14.3	2	9.2
Number of ANC visits						
<4	69	11.0	7	10.1	6	8.7
4-7	290	46.0	28	9.7	16	5.5
≥ 8^b^	271	43.0	22	8.1	10	3.4

a = One missing,

b = Ghana's standard for antenatal care visits,

* = Statistically significant (p ≤ 0.05)

**Table 2:** Preventive services offered during antenatal care visits, Kumasi 2011

Predictors	N = 630			Malaria Yes = 57				Helminths = 32		

	N	%	n	%	aOR	95% CI	*n*	(%)	aOR	95% CI
Malaria prophylaxis					1.98	(0.92-4.28)	NA			
Yes	571	90.6	48	8.4						
No	59	9.4	9	15.3						
IPT doses^a^							NA			
0	99	15.7	15	15.2	2.10	(1.06-4.17)*				
1	80	12.6	4	5.0	0.62	(0.21-1.82)				
2	130	20.6	13	10.0	1.33	(0.66-2.70)				
3	321	50.9	25	7.8	ref					
Bed net use					0.90	(0.52-1.57)	NA			
Yes	240	38.1	23	9.6						
No	390	61.9	34	8.7						
Helminth screening			NA						2.43	(1.15-5.12)*
Yes	479	76.0					19	4.0		
No	151	24.0					13	8.6		
Antihelmintics			NA						0.76	(0.34-1.70)
Yes	149	23.6					9	6.0		
No	481	76.3					23	4.8		

a Fishers Exact test,

* = Statistically significant (p ≤ 0.05), aOR = Adjusted for recruiting site, NA = Not applicable, IPT = Number of times intermittent preventive treatment of antimalarial drug (SP) taken

**Table 3:** Anemia and pregnancy outcomes by infection status

Predictors	N = 630		Malaria Yes = 57				Helminths = 32			

	N	%	n	%	aOR	95% CI	*n*	%	aOR	95% CI
Severe/moderate (Hb<8 g/dL)	53	8.4	7	13.2	1.65	(0.68-4.00)	4	7.6	1.66	(0.53-5.17)
Mild (Hb 8-11g/dL)	241	38.3	22	9.1	1.13	(0.63-2.03)	12	5.0	1.03	(0.48-2.22)
Normal (Hb> 11 g/dL)	336	53.3	28	8.3			16	4.8		
Adverse outcome					1.34	(0.75-2.55)			1.68	(0.77-3.65)
Yes	140	22.2	16	11.4			10	7.1		
No	490	77.8	41	8.4			22	4.5		

Hb = Haemoglobin level, aOR = Adjusted for recruiting site

**Table 4:** Crude and adjusted odd ratios of malaria and helminths infections and adverse birth outcomes of 630 women in Kumasi, 2011

Infections status	Any adverse birth outcome		Low birth weight		Preterm		Stillbirth	

	OR (95% CI)	aOR (95% CI)	OR (95% CI)	aOR (95% CI)	OR (95% CI)	aOR (95% CI)	OR (95% CI)	aOR (95% CI)
Malaria	1.4 (.78-2.60)	1.3 (.66-2.38)	1.3 (.56-2.99)	1.0 (.41-2.52)	1.6 (.70-3.83)	1.7 (.70-3.88)	1.0 (.36-3.00)	1.0 (.34-2.96)
All Helminths	1.6 (.76-3.54)	1.7 (.75-3.66)	2.2 (.86-5.54)	2.1 (.80-5.14)	1.6 (.55-4.86)	1.6 (.54-4.91)	1.4 (.42-4.94)	1.6 (.44-5.51)
Helminth types								
Blood suckers^a^	3.6(1.03-12.63)*	3.9(1.09-14.20)*	4.1(1.02-16.17)*	4.03(0.96-16.89)	2.87(0.59-13.87)	3.29(0.67-16.22)	6.0(1.49-24.00)*	7.7(1.21-36.38)*
Non-blood suckers	1.1 (.38-2.92)	1.1 (.36-2.92)	1.5 (.43-5.22)	1.4 (.38-5.03)	1.2 (.26-5.05)	1.1 (.23-4.78)	0.5 (.0-2.11)	0.4 (0-2.09)
Malaria+ Helminths	7.1 (.64-78.55)	4.7 (.40-55.73)	4.6 (.41-50.97)	3.5 (.27-44.93)	5.7 (.50-63.38)	4.9 (.40-59.91)	3.6 (0-23.70)	2.0 (0-14.08)

OR = crude odd ratios, aOR = Adjusted for HIV status, haemoglobin concentration, age and parity (primipara or multipara)

a Helminths associated with blood loss (hookworm and blood fluke),

* = Statistically significant (p-value ≤ 0.05)
